# Fistula upon the Face

**Published:** 1881-12

**Authors:** Eugene S. Talbot

**Affiliations:** Clinical Lecturer on Dental and Oral Surgery at Central Free Dispensary, Rush Medical College


### Clinical Reports.

ARTICLE V.

### FISTULA UPON THE FACE. By EUGENE S. TALBOT, M.D., D.D.S. Clinical Lecturer on Dental and Oral Surgery at Central Free Dispensary, Rush Medical College. Reported by J. LONG.

This patient, Miss Sarah K——, has kindly consented to appear before you, in order to furnish an opportunity of studying an important lesion. I trust her case will so impress itself upon your minds as to assist you in treating like cases intelligently.

This young woman is an American, sixteen years of age, and in perfect health. Her parents are both living and healthy. She has been suffering from this trouble six years. It first manifested itself while the patient was living in New Orleans, by pain and swelling on the side of the face. A physician was summoned, who made an incision at the border of the jaw, anterior to the facial artery, where he had discovered pus. The pus discharged itself in large quantities, but the wound did not heal; pus constantly trickled out of the opening, and a permanent fistula was established. Three years afterward the family moved to Chicago, and the girl was treated by a physician who probed and burnt it at intervals for two years. At the end of that time, there being no improvement, she went to one of our hospitals, and while under the influence of ether an operation was performed, and the wound sewed up. When this failed to heal, she applied to a physician, who assured her the previous treatment was hasty; that she was scrofulous, and needed constitutional treatment. Last June, while under his care, she came into my office with a patient of mine. As I was about to dismiss my patient, my attention was called to her friend's disfigured face.

I requested her to open her mouth. Upon doing so, I found only the roots of the first and second molars posterior to the bicuspids, upon the right side of the inferior maxilla. I informed her that a cure could be effected by a removal of the roots. As she had not prepared herself for an operation, she postponed it for a time.

Two weeks ago she appeared again, and desired me to take the roots out. Upon removing the anterior root of the second molar, I found an abscess, the cause of all her suffering. You observe that the fistula has now entirely healed. A swollen face is indicative of bad teeth, so we can presume this trouble proceeds from the teeth, before further considering the condition of the health.

Observe this ugly contracted scar upon the border of the jaws, and question yourselves if it is ever justifiable to puncture an abscess upon the face. After ascertaining the offending tooth, if badly decayed, or a root, remove it, that the abscess may discharge itself through the opening in the jaw. If, for any reason, you should wish to lance the abscess first, open the mouth and draw the cheek out with the finger, then pass your bistoury between the mucous membrane and the bone, the blade resting against the jaw, thus avoiding the facial artery. The pus will pass into the mouth, and prevent the scarring of the face. Should a fistula be already established upon the face, examine the mouth, and if you succeed in finding the seat of the lesion, remove the tooth or root, and nature will heal the wound.

---

BEACONSFIELD'S DOCTORS.—Dr. Quinn has been called upon to defend himself before the College of Physicians for consulting with Dr. Kidd, who is an eclectic practitioner. Dr. Quinn explained that before seeing Lord Beaconsfield, he received a letter from Dr. Kidd, saying that he was not treating the case homœopathically, and that every direction and prescription of Dr. Quinn's would be faithfully carried out. From a letter of Dr. Kidd to the medical journals it appears that Sir William Jenner absolutely refused a consultation prior to the summoning of Dr. Quinn.—*Medical News.*

